# Distinct Features of Proton-Induced X-ray Emission (PIXE) and Inductively Coupled Plasma Mass Spectrometry (ICP-MS) When Used for Elemental Analysis of Nail Samples

**DOI:** 10.1007/s12012-025-10011-9

**Published:** 2025-05-16

**Authors:** Kristina M. Zierold, Melissa J. Smith, Jason Xu, Lu Cai, Lonnie Sears

**Affiliations:** 1 Department of Environmental Health Sciences, School of Public Health, University of Alabama at Birmingham, RPHB 534 C, 1720 2nd Ave S, Birmingham, AL 35294-0022 USA; 2 Department of Biostatistics, School of Public Health, University of Alabama at Birmingham, Birmingham, AL USA; 3 Department of Pediatrics, Pediatric Research Institute, University of Louisville School of Medicine, Louisville, KY USA; 4 Departments of Radiation Oncology, Pharmacology and Toxicology, University of Louisville School of Medicine, Louisville, KY USA; 5 The Center for Integrative Environmental Health Sciences, University of Louisville School of Medicine, Louisville, KY USA; 6 Department of Pediatrics, University of Louisville School of Medicine, Louisville, KY USA

**Keywords:** Biomarkers, PIXE, ICP-MS, Lin’s concordance correlation coefficient, Health studies

## Abstract

Biomarkers, such as toenails, are commonly used to investigate the health status of individuals. Nails samples are a useful marker of exposure, as they are easy to collect, store, and represent exposure from 6 to 12 months. There are multiple analytical methods that can be used to extract long-term exposure profiles from toenails including Proton-Induced X-Ray Emission(PIXE) and Inductively Coupled Plasma Mass Spectrometry (ICP-MS). The overall goal of this research was to evaluate the level of agreement between the two analytical methods for assessment of different metals in nail samples. Children’s nail samples were collected. Nail samples were first analyzed by PIXE and then analyzed by ICP-MS. To compute quantiles for the metal concentrations that had some fully observed and some left-censored concentrations, a reverse Kaplan–Meier estimator was used. Lin’s concordance correlation coefficient (CCC) and the Pearson correlation coefficient were calculated to assess agreement between the two methods and to determine the strength of the linear association between the metal concentration measurements obtained under each analytical technique. PIXE and ICP-MS determined similar median concentrations for calcium, copper, potassium, and nickel. However, there were stark differences between other elements. Several elements, such as copper, potassium, and zinc represented strong concordance through use of the CCC. In many studies, scholars want to evaluate how well one measurement can reproduce another, and our paper used several elements to show the degree of reproducibility between the two analytical methods. This can be useful when scholars are determining methods to assess biomarkers in health-related studies.

## Introduction

Cardiovascular diseases (CVDs) are the leading causes of death throughout the world [1, 2]. Globally, ischemic heart disease was the leading cause of CVD mortality with an aged-standardized rate of 108.8 deaths per 100,000 [3]. Environmental exposures have been associated with CVDs. Exposure to increased concentrations of metals has been associated with CVDs in both animal models and epidemiological research [4–11]. Chowdhury et al. (2018) compared the top versus the bottom thirds of baseline copper levels and found that there were significant aggregated relative risks for CVDs, including coronary heart disease. Additionally, the authors found arsenic, lead, and cadmium significantly increasing the risk [10]. Martinez-Morata (2024) reported a significant association of the CVD incidence with cadmium, copper, and zinc [11].

Researchers investigating exposures to metals with health outcomes frequently involve the use of biomarkers such as hair or toenails, or fingernails. Toenails and fingernails are commonly used as biomarkers for metals because they bind to the keratin in the nail and represent long-term exposure (3–12 months) [12, 13]. Nail samples are less invasive to collect compared to urine or blood samples and do not need special methods of storage [9–13]. Research has shown that concentrations of metals and other elements are greater than other biomarkers because they are not exposed to metabolic processes [12, 14]. Furthermore, multiple analytical techniques such as Particle Induced X-ray Emissions (PIXE) and Inductively Coupled Plasma Mass Spectrometry (ICP-MS) can be used to analyze the concentrations of the elements in nails.

PIXE is an X-ray spectroscopic technique that can be used to analyze various media. It is a non-destructive method which analyzes the whole sample allowing for simultaneous elemental analysis of 72 inorganic elements (sodium to uranium) [15, 16]. PIXE requires a single run and provides good precision and accuracy (ranging between 5 and 15%) [17, 18]. A thin proton beam is required for the PIXE analysis to occur. Prior to the vacuum chamber, an accelerator tube is used to remove electrons from the elements, leaving positively charged ions. From these ions, a proton beam is formed, which is then adjusted to bombard the center of the sample. As the proton beam hits the sample, inner shell electrons (K, L shells) in atoms of the elements become excited, are expelled, and vacate the shells. These vacancies are then filled by electrons from higher level shells (L, M, N, etc.). Production of X-rays occurs when the higher shelled electrons drop into the vacancies [19, 20]. The energies of the X-rays are exclusively characteristic of the elements in the sample [16]. The number of X-rays that are emitted at a given energy level corresponds to the mass of the elements being assessed in the sample [16, 21].

ICP-MS is commonly used to analyze liquid samples or samples that can be dissolved, or acid digested, to generate a liquid. The liquid sample after it is nebulized is swept into an argon plasma which dissolves, vaporizes, atomizes and ionizes [22, 23]. The stream of ionized atoms is then sent through a mass filtering device (mass spectrometer), where the ions strike a first dynode of an electron multiplier (EM). After hitting the first dynode, ions emit electrons which hit a second dynode, for which more electrons are released. As this process continues, it culminates into a signal pulse, which is compared to standards, that allows for the development of a calibration curve that determines the concentration of the element [22]. Therefore, ICP-MS can measure virtually every naturally occurring element plus many non-natural “radiogenic” isotopes such as technetium, neptunium, plutonium, and americium. The only elements that ICP-MS cannot measure are hydrogen (H) and Helium (He) (which are below the mass range of the mass spectrometer), argon (Ar), nitrogen (N), and oxygen (O) (which are present at high level from the plasma and air), and fluorine (F) and neon (Ne) (which cannot be ionized in an Ar plasma) [23].

The objective of this study was to conduct an intercomparison of two analytical methods (PIXE and ICP-MS) to evaluate the level of agreement between the methods for different metals in nail samples that were collected from children. It may also provide useful information for researchers utilizing these techniques when working with nail samples as biomarkers.

## Materials and Methods

### Collection of Toenail/Fingernail Samples

Toenails and fingernails were collected from 235 children aged 6–14 years old living within 10 miles of the centroid between the two coal-fired power plants in Jefferson and Bullitt Counties in Kentucky [24]. A minimum of 150 mg was collected from each child. The nails were cut using stainless steel nail clippers, by the child or the parent/guardian and stored in plastic containers.

Before sending the nail samples to the laboratory for analysis, each child’s nails were washed and dried. Nail samples were cleaned in a two-step process. The first step involved using the agitation of an acetone rinse, which was then followed by two agitated rinses with deionized water. This cleaning process was used to remove surface dirt and nail polish, without altering the elemental composition of the nails. The nails were set to dry and then placed into a clean plastic container.

### PIXE Preparation and Method

#### Nail Sample Preparation

The children’s nail samples were delivered to and analyzed by Elemental Analysis Inc. (EAI) in Lexington Kentucky. Prior to the analysis, each individual child’s nails were cryogenically frozen, ground and mixed with a neutral binding agent. The nail mixture of each child’s sample was then made into a 5/8 inch diameter pellet for the PIXE analysis.

#### PIXE

PIXE measurements were made using a 3 million electron volt (MeV) proton beam that was generated by a General Ionex 4 mega volts (MV) tandem accelerator with a duoplasmatron source that produced a beam current 0.5 amps for our samples. Protons from the accelerator entered the target chamber via a 0.30 mil Kapton window. The target chamber fits a carousel projector slide mechanism that can hold 80 slides and a linear tracking mechanism, with height correction 20.3 cm of horizontal travel and a stepping resolution of 0.01 mm [25].

X-rays exit through a 0.1 mil Mylar window which is at 45° relative to the beam axis. A 30 mm^2^ Si (Li) detector was used in two positions for X-ray collection; a close-in and a back-out position. Using two detector positions provided analysis of the high and lower end of the X-ray spectrum.

To determine the elemental concentrations, the X-ray spectrum was analyzed using the GUPIX software developed by the University of Guelph and modified specifically for EAI [26]. Reports that were sent to the research team included the appropriate element name or symbol, the energy (kEV) of the x-ray line used to quantify the element, the detection limit expressed as a weight fraction of the sample, and the weight fraction of the element present in the sample expressed as either % or ppm.

### ICP-MS Preparation and Method

#### Nail Sample Preparation

Children’s toenail samples were collected in plastic containers and stored in dry place. Ten to 15 mg toenail sample was weighted and transferred to 2 ml acid pretreated digestion tube (#1420–9399, USA Scientific), then 300 µl 70% nitric acid (trace metal grade, Fisher Scientific Cat# A509-P500) was added to the sample tube. Toenail samples were digested in 65 °C shaker for 4 h until the solution became clear. After that 100 µl H_2_O_2_ (Sigma Cat# 95,321) was added to each sample for further 2 h digestion. Digested samples were transferred to working hood and cooled down to room temperature then diluted all solution to 4 ml DI water (Millipore, Milli Q Academic), finally assay volume was 4.4 ml.

#### Mass Spectrometry

To measure the metal content in the child’s nail samples, Agilent 7800 ICP-MS (Inductively Coupled Plasma Quadrupole Mass Spectrometer, Agilent Technologies, Japan) was used. This Agilent 7800 ICP-MS was optimized by performance check with 1 ppb tuning solution and assay program was auto tuned by 10 ppb tuning solution (Agilent Cat#5188–6564). The auto sampler SPS 4 was used for sample introduction. 25 metals calibration standard was purchased from Inorganic Ventures (Cat# IV-STOCK-50) and serial metal standard dilutions were made with same acid matrix of samples. Internal standard was purchased from Agilent (Cat#5188–6525). Assay program was run by Agilent MassHunter software with He mode and each sample was read three times for final mean value.

### Statistical Analysis

There were 235 children that provided nail samples in the study. To be included in the study, children must have had at least one PIXE measurement or PIXE limit of detection (LOD) recorded and at least one ICP-MS measurement in the dataset. The mean age of the children in the study was 11 years old and the sample was 53% male, and 75% white. All statistical analyses were performed using version 4.2.2 of the R statistical software.

The distributions of the elemental concentrations obtained using the PIXE and ICP-MS analytical methods were summarized by estimating and comparing the 25 th, 50 th, and 75 th percentiles for each metal. No metal concentrations fell below the LODs using the ICP-MS method, so each of these quantiles were computed using the observed concentrations. Similarly, when 100% of the PIXE concentrations were above the LOD, this method was used to compute the 25 th, 50 th, and 75 th percentiles of the metal concentrations determined by PIXE.

For certain metals, the PIXE concentrations were available for some children, whereas the concentrations fell below the LODs for other children. To compute quantiles for the metals that had some fully observed and some left-censored concentrations, a reverse Kaplan–Meier estimator was used [27]. The reverse Kaplan–Meier method estimates the cumulative distribution function, and in turn the quantiles, of the concentrations of the metal when some measurements fall below the LOD. It makes use of both the fully observed data values and the partial information provided by the LODs (i.e., it is known that the concentration falls somewhere between 0 ppm and the LOD). The reverse Kaplan–Meier estimator assumes random censoring, meaning that the LOD is not related to the unobserved value of the metal concentration. Since the LOD measurements are based on the analytical technique for PIXE, this assumption was deemed reasonable. The NADA2 and EnvStats packages in R were used to obtain quantiles from the reverse Kaplan–Meier estimator. Quantiles were estimated for all 27 metals that were measured by both PIXE and ICP-MS, as long as at least 25% of concentrations fell above the LOD. The percentage above the LOD for each of the 71 metals analyzed by PIXE was also calculated.

In addition, Lin’s concordance correlation coefficient (CCC) and the Pearson correlation coefficients were calculated to assess agreement and the linear associations under each analytical technique. Nine metals were included in the CCC analysis, as we stopped reporting % above LODs at nickel (Ni) as the other elements had percentages that were below 70%. The CCC is a correlation coefficient specifically designed to assess agreement, or how closely the points on the scatterplot (with one technique on the x-axis and the other technique on the y-axis) fall to the one-to-one line combined with how strong the linear relationship is between ICP-MS and PIXE concentrations [28]. A CCC of 1 indicates perfect concordance between the log concentrations. In contrast, the Pearson correlation coefficient indicates the strength of the linear relationship between the log concentrations measured under the ICP-MS and PIXE measurements but does not detect if the actual values are in alignment.

The metal concentrations were log-transformed because the distributions were highly skewed with apparent outliers. To compute 95% confidence intervals for the CCC and the Pearson correlation coefficient, the assumption of multivariate normality between the two sets of measurements is required [28]; under the log transformation, it was reasonable to make this assumption, whereas this assumption was not reasonable using the raw data. Both point estimates and 95% confidence intervals were calculated for each of these coefficients to numerically summarize the overall agreement and strength of linear association between ICP-MS and PIXE. Finally, the level of agreement between the two techniques was visually compared by creating scatterplots with the log (PIXE concentration) on the x-axis and the log (ICP-MS) concentration on the y-axis. A one-to-one line was included. PIXE concentrations that fell below the LOD were indicted with an X to illustrate that these observations were left-censored.

## Results

As an exploratory analysis, we were interested in obtaining measures of the elements PIXE analysis could determine. However, PIXE was unable to determine concentrations of many of the elements in our samples, because the LODs were too high. Table 1 reports the percent of elements above the LOD based on the PIXE analysis.

**Table 1:** Percentage of PIXE measurements above the LOD for each element

Element	% above LOD	Element	% above LOD	Element	% Above LOD
Ca	100.00	Ga	0.85	Pd	0.00
Cl	100.00	Hg	0.85	Pm	0.00
Cu	100.00	Mo	0.85	Pr	0.00
Fe	100.00	Ag	0.43	Pt	0.00
K	100.00	Ce	0.43	Re	0.00
S	100.00	Er	0.43	Ru	0.00
Zn	100.00	Au	0.00	Sb	0.00
Si	99.15	Ba	0.00	Sc	0.00
Na	97.87	Cd	0.00	Sm	0.00
P	97.87	Co	0.00	Ta	0.00
Mg	95.32	Cs	0.00	Tb	0.00
Al	87.23	Dy	0.00	Tc	0.00
Ni	69.36	Eu	0.00	Te	0.00
Br	68.51	Gd	0.00	Th	0.00
Cr	55.32	Ge	0.00	Tl	0.00
Ti	46.81	Hf	0.00	Tm	0.00
Zr	28.09	Ho	0.00	U	0.00
Mn	20.43	I	0.00	V	0.00
Se	10.64	In	0.00	W	0.00
Sn	8.09	Ir	0.00	Y	0.00
As	6.81	La	0.00	Yb	0.00
Sr	5.96	Lu	0.00		
Rb	2.55	Nb	0.00		
Pb	1.70	Nd	0.00		
Bi	1.28	Os	0.00		

Unlike PIXE, ICP-MS analysis was able to determine concentrations of many elements [23], and detected 27 elements (sodium (Na), magnesium (Mg), aluminum (Al), potassium (K), calcium (Ca), titanium (Ti), vanadium (V), chromium (Cr), manganese (Mn), iron (Fe), cobalt (Co), nickel (Ni), copper (Cu), zinc (Zn), arsenic (As), selenium (Se), zirconium (Zr), molybdenum (Mo), silver (Ag), cadmium (Cd), tin (Si), antimony (Sb), barium (Ba), mercury (Hg), thallium (Tl), lead (Pb), thorium (Th), and uranium (U)) in the nail samples. Table 2 reports the elemental concentrations from the two analytical methods.

**Table 2:** Elemental concentrations observed for PIXE and ICP-MS analysis

Element	PIXE				ICP-MS		
	% Above LOD	Q1	Median	Q3	Q1	Median	Q3
Ca	100.00	618.25	762.16	920.04	622.60	752.13	936.90
Cu	100.00	3.70	5.04	6.59	4.31	5.18	6.95
Fe	100.00	48.35	67.88	107.50	20.41	31.51	46.12
K	100.00	481.38	783.76	1190.00	486.12	793.60	1203.50
Zn	100.00	75.96	86.00	97.16	88.70	101.07	112.82
Na	97.87	1080.00	1520.00	2140.00	691.51	1017.01	1461.49
Mg	95.32	212.60	255.30	308.00	98.41	115.52	140.03
Al	87.23	95.16	139.00	205.60	13.17	19.48	29.61
Ni	69.36	< 0.41	1.45	2.60	0.78	1.40	2.84
Cr	55.32	< 1.12	4.00	6.70	0.61	0.98	1.81
Zr	28.09	< 1.13	< 1.13	< 6.05	1.33	2.14	4.02
Mn	20.43	*	*	*	0.58	0.96	1.72
Se	10.64	*	*	*	0.52	0.61	0.72
Sn	8.09	*	*	*	0.22	0.38	0.77
As	6.81	*	*	*	0.07	0.11	0.21
Pb	1.70	*	*	*	0.27	0.47	0.84
Hg	0.85	*	*	*	0.01	0.02	0.04
Mo	0.85	*	*	*	0.03	0.04	0.07
Ag	0.43	*	*	*	0.03	0.05	0.11
Ba	0.00	*	*	*	0.82	1.16	1.92
Cd	0.00	*	*	*	0.02	0.04	0.07
Co	0.00	*	*	*	0.02	0.03	0.05
Sb	0.00	*	*	*	0.07	0.12	0.18
Th	0.00	*	*	*	0.00	0.00	0.01
Tl	0.00	*	*	*	0.00	0.00	0.00
U	0.00	*	*	*	0.00	0.01	0.01
V	0.00	*	*	*	0.02	0.03	0.05

^*^Did not estimate as less than 25% of the observations fell below the LOD

These were computed using all data (left-censored and fully observed) and accounts for LODs. Only PIXE elements also analyzed by ICP-MS are included

Among the elemental concentrations in Table 2, PIXE and ICP-MS determined similar median concentrations and Q1, Q3 values for Ca, Cu, K, and Ni. However, there were stark differences between the elemental concentrations of Fe, Mg, and Al. Elemental concentrations that were determined by PIXE analysis were approximately twice the median value for Fe and Mg and seven times the median value for Al, compared with the elemental concentrations determined by ICP-MS analysis. The median concentrations of PIXE analysis of Zn (86 µg/m^3^) and Na (1520 µg/m^3^) were not remarkably different than the ICP-MS (101 µg/m^3^,1,017 µg/m^3^) determined values.

**Fig. 1:**
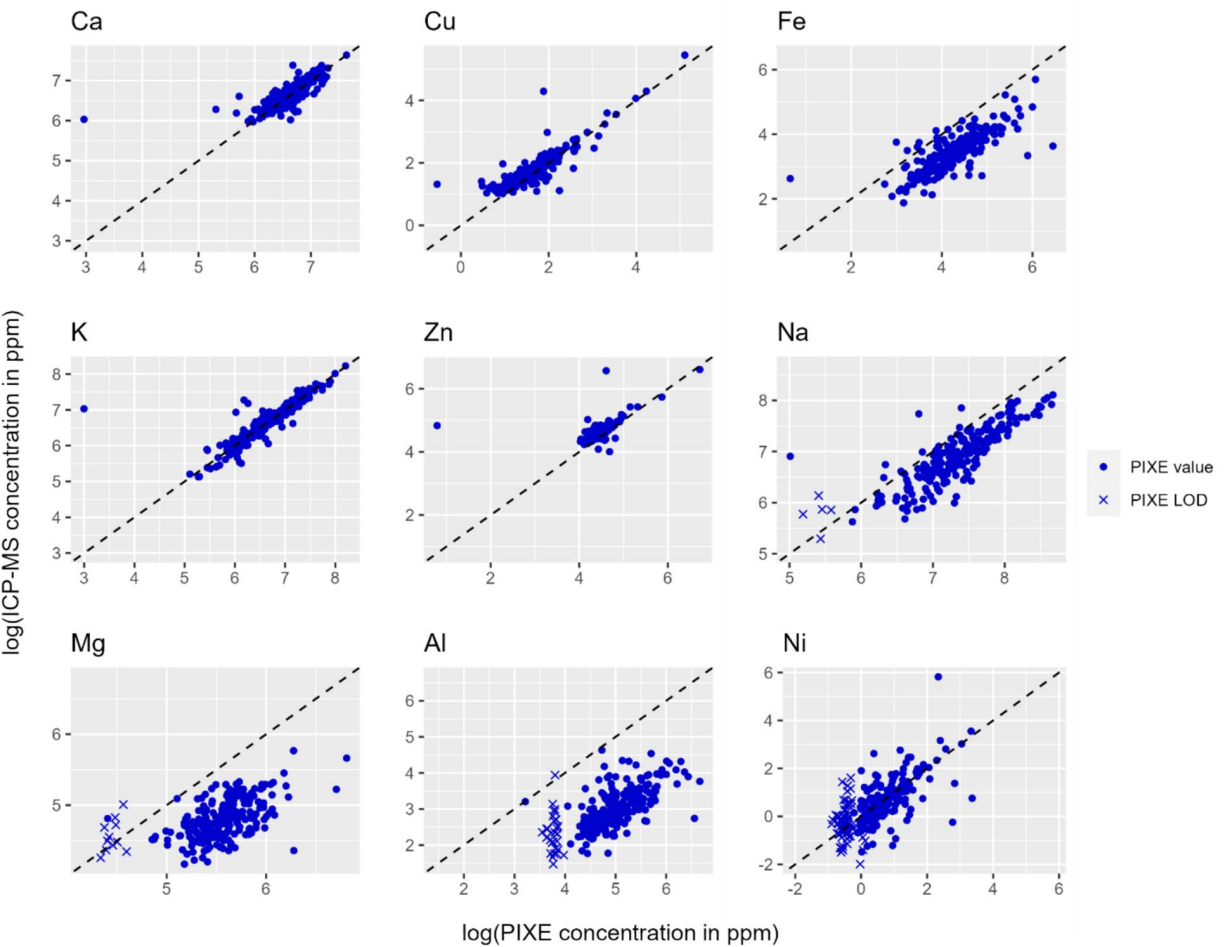
Scatterplots of concordance between elements analyzed by ICP-MS and PIXE

Figure 1 provides scatterplots of the relationships between the log-transformed elemental concentrations from ICP-MS and PIXE. The dotted lines indicate perfect concordance (agreement) between the log concentrations using PIXE and using ICP-MS. If a point on the scatterplot is marked with an “X,” this represents that the PIXE measure is left-censored, meaning that the concentration is below the LOD. For those points with X’s, we used the elemental LOD as the number plotted on the x-axis. This suggests that the true value of the log concentration measured under PIXE would have been lower, and the true point on the scatterplot would be located further to the left.

Figure 1 presents the relationships between PIXE and ICP-MS analyses. Ca, Cu, and Zn, and K, represents strong concordance (fall close to the 1–1 line), Although Ni appears to have good concordance between PIXE and ICP-recall as displayed in Table 1, PIXE only had 69% of measurements above the LOD (as shown by the x on the plot). Like Ni, any element that has many LODs from PIXE, we cannot determine its exact PIXE concentration, but we know points would be to left, not to right and would be lower. Mg and Al show poor similarity in the data from PIXE and ICP-MS.

Table 3 reports correlation coefficient estimates and 95% confidence intervals (CI) for CCC analysis and Pearson correlation analysis. The Pearson correlation coefficient represents the strength of the linear relationship between log (PIXE) and log (ICP-MS) concentrations, with values closer to one indicating a stronger linear relationship between log concentrations determined using the two methods. The CCC estimate quantifies agreement in the values and measures of deviations from the 1–1 line in the scatterplots reported in Fig. 1. A CCC of one indicates no deviation from the 1–1 line. The lower the CCC, the less similar are the toenail concentrations measured by PIXE and ICP-MS. The CCC method cannot account for censoring, therefore only the elements that had a very high percentage above the LOD were included in Table 3.

**Table 3:** Correlation Estimates and 95% CI from CCC and Pearson Analyses

Element	CCC estimate	CCC 95% CI	Pearson correlation estimate	Pearson correlation 95% CI
Ca	0.70	(0.64, 0.76)	0.73	(0.67, 0.79)
Cu	0.84	(0.80, 0.87)	0.86	(0.83, 0.89)
Fe	0.41	(0.35, 0.47)	0.76	(0.71, 0.81)
K	0.86	(0.82, 0.89)	0.86	(0.82, 0.89)
Zn	0.45	(0.36, 0.54)	0.54	(0.45, 0.63)

By looking at Table 3, we can see that log(K) and log(Cu)are in excellent agreement with each other. For log(K), the CCC is 0.86 (95% = 0.82, 0.89) and for log(Cu) the CCC is 0.84 (95% = 0.80, 0.87). In addition, log(K) and log(Cu) show very strong Pearson Correlation Estimates. These findings suggest that the CCC of these two measures in toenails represent excellent agreement when using PIXE or ICP-MS and also show a strong linear relationship. While not excellent, Log(Ca) shows a strong relationship as well (CCC = 0.70, Pearson = 0.73).

There is less agreement between the other elements’ log concentrations when measured with PIXE and ICP-MS (Fig. 1 and Table 2 show the agreement is poor in some cases). For example, the CCC for Fe is 0.41 and for Zn, it is 0.45, indicating that that PIXE and ICP-MS do not represent similarity between the two sets of toenail data. Interesting there is a strong linear relationship between the log (Fe) measured with PIXE (0.76, 95% CI = 0.71, 0.81) but the agreement between the PIXE and ICP-MS is poor (CCC = 0.41, 95% CI = 0.35, 0.47).

## Discussion

In this paper, we assessed the agreement/concordance between elemental measurements from nail samples of children. The elements were assessed by two analytical methods (PIXE and ICP-MS) and using Lin’s CCC. In many studies, scholars want to evaluate how well one measurement can reproduce another, and this was the intent of our paper. The CCC can also be used to determine a new method compared to a gold standard.

Our findings indicate that there is an excellent agreement in PIXE and ICP-MS for the elemental concentrations of log (Cu) and log (Mg). Log (Ca) shows a strong relationship as well. The other elements do not agree well, meaning the reproducibility of these measurements is not strong between PIXE and ICP-MS. In fact, this inconsistence for Fe measurement between PIXE and ICP-MS was reported previously [29]. They measured components of urban particulate matter (PM), Buffalo River sediment, and pine needles with three methods: PIXE, ICP-MS, and ICP-AES (atomic emission spectrometry). In general, most of values measured by three methods are consistent and close among three kinds of samples, but there were also values inconsistent among three methods dependent on samples. For instance, measurements of pine needles with PIXE, ICP-MS, and ICP-AES showed ICP-MS with lowest Fe and Al concentrations. The low concentrations of Fe and Al with ICP-MS are consistent with the finding in the present study Therefore, more studies to compare the reproducibility of these measurements different kinds of samples are urgently needed to better guidance for the choice of different assays to measure metals under different projects. We used the Pearson Correlation as a comparison measure because some readers may not be familiar with the CCC. However, it may be best to focus on CCC when both the PIXE and ICP-MS measurements are similar. When they are different, like for Fe which has a low CCC but high Pearson correlation, this illustrates the limitations of using Pearson correlation for agreement studies. CCC picks up both the linear relationship and how similar the values are to one another. The Pearson correlation suggests that we could pretty accurately predict the value of ICP-MS from PIXE (or vice-versa) using a linear relationship, but the values aren’t the same.

Compared to the CCC, there are several short-comings when using other statistical methods to assess agreement/reproducibility [30]. Some of these short-comings include issues with paired t-tests. When the variance is small, the paired t-test can reject a strong agreement between the two approaches. And if the pairs of data have equal means, the paired t-test can fail to detect poor agreement.

One limitation of the CCC is that there is not clear agreement on how to interpret the findings. Most scholars have thus used the Pearson Correlation Values as a way to understand how similar the values are [31]. A strength to using the CCC, is that is can be used for small samples sizes as we had in our study.

To date, although methods used to measure metals in the biological samples for investigating cardiovascular diseases were diverse, the ICP-MS has been mostly used in the available studies. For example, Zhang et al. used PIXE to show the decreased Cu level in the heart of diabetic rats associated with the cardiac dysfunction, while Liu et al. used graphite furnace atomic absorption spectrophotometry investigated the association of TAC-induced cardiac hypertrophy with low levels of cardiac Cu [32]. However, many studies on cardiac toxicity induced by aging, anticancer drugs, and diabetes used ICP-MS assay for measuring cardiac metal levels of human [33–36] and animal samples [37–40]. Even though there was one study on the deposition of heavy metals in biological tissues (hair and nails) of workers in metal workshops with both ICP-MS and PIXE, they measured metals in the particulate matter (PM) samples at the workplace with PIXE and measured metals in the biological tissues with ICP-MS [41].

PIXE and ICP-MS both have limitations with elemental analysis of thick biomarkers, such as the nail samples used in this research. PIXE allows for a 72 elemental analysis using non-destructive method, making it well suited for experimental situations with unknown elements. However, for low particle energies and lower atomic number elements (Z < 12), PIXE may inaccurately quantify elements because of background noise and the sample concentration and density of the matrix. Additionally, unless low beam currents are used, deterioration of the sample may occur. Low beam currents result in low sensitivity [42].

ICP-MS often considered the “gold standard” for elemental analysis in biomarkers, is a destructive technique that has the ability to quantify elemental concentrations in the parts per billion (ppb) to parts per trillion (ppt) range [43, 44]. It requires long and careful sample preparation to avoid contamination and the method does not analyze light elements (Z < 7). ICP-MS can be sensitive to polyatomic ions that can cause spectroscopic interference. Polyatomic ions often result from reagents used in sample preparation or are derived from the sample matrix. Metals that are located in the fourth row of the periodic table (e.g., arsenic, manganese) commonly overlap with polyatomic ions, potentially causing inaccurate measurements [43]. Another method of spectroscopic interference that may affect accurate concentration measures arise from analysis of isobaric elements. Elements that are isobaric have different atomic numbers, but the same mass number. Many metals are isobaric (e.g., nickel, cadmium, chromium) and must be corrected by using mathematical models or using a reactive gas such as oxygen [43].

Although there are benefits and limitations of both ICP-MS and PIXE, both have great potential for use in assessing elemental concentrations. The intent of this research was to do an intercomparison of PIXE and ICP-MS when assessing nail samples. Although this paper does not suggest the “best” method to use for analyzing nails, it can be used to understand which method offers the strongest potential for reproducibility. Further studies are warranted to investigate the metals we could not, because of low LODs in many of the nail samples.

## Data Availability

The data provided in this manuscript is confidential and only available by contacting the first author. The data may or may not be shared.
